# New xanthone and chemical constituents from the aerial parts of *Mallotus glomerulatus* and their cytotoxicity in MCF-7 and MDA-MB-231 breast cancer cells

**DOI:** 10.5599/admet.2901

**Published:** 2025-09-21

**Authors:** Napason Chabang, Chanikarn Wongwitayasombat, Patoomratana Tuchinda, Bamroong Munyoo, Niwat Kangwanrangsan, Suradej Hongeng, Bodee Nutho, Sitthivut Charoensutthivarakul, Phongthon Kanjanasirirat

**Affiliations:** 1 School of Bioinnovation and Bio-based Product Intelligence, Faculty of Science, Mahidol University, Bangkok, Thailand; 2 Biomedical Science Program, Faculty of Science, Mahidol University, Bangkok, Thailand; 3 Excellent Center for Drug Discovery, Faculty of Science, Mahidol University, Bangkok, Thailand; 4 Department of Pathobiology, Faculty of Science, Mahidol University, Bangkok, Thailand; 5 Department of Pediatrics, Faculty of Medicine Ramathibodi Hospital, Mahidol University, Bangkok, Thailand; 6 Department of Pharmacology, Faculty of Science, Mahidol University, Bangkok, Thailand; 7 Center for Neuroscience, Faculty of Science, Mahidol University, Bangkok, Thailand

**Keywords:** Cleistanthin A, xanthone, breast cancer, V-ATPase

## Abstract

**Background and purpose:**

Breast cancer remains a significant global health burden, especially in low-resource settings where standard therapies are limited. This study aimed to explore *Mallotus glomerulatus*, a lesser-known Thai medicinal plant, as a potential source of novel anti-breast cancer agents.

**Experimental approach:**

A phytochemical investigation of *M. glomerulatus* resulted in the isolation and structural characterization of a novel xanthone (Compound **1**) and cleistanthin A (Compound **10**) using UV, IR, NMR, and HRMS techniques. Cytotoxicity of the compounds was evaluated in vitro against MCF-7 (ER-positive) and MDA-MB-231 (triple-negative) breast cancer cell lines, along with HepG2 liver cells. Molecular docking studies were conducted to assess their interaction with vacuolar H^+^-ATPase (V-ATPase).

**Key results:**

Compound **1** demonstrated selective cytotoxicity toward MCF-7 cells, whereas cleistanthin A exhibited potent cytotoxicity against both breast cancer lines, with nanomolar IC_50_ values and a high selectivity index (>100) for MDA-MB-231 compared to HepG2 cells. Docking analysis revealed favourable binding of both compounds at the a–c subunit interface of V-ATPase, suggesting a mechanism involving proton pump inhibition and lysosomal dysfunction.

**Conclusion:**

The findings highlight *M. glomerulatus*, particularly cleistanthin A, as a promising source of safe and affordable anti-breast cancer compounds with potential therapeutic value. Further studies on the mechanism and *in vivo* efficacy are warranted.

## Introduction

Breast cancer is the most common type of cancer with the highest incidence and lethality rate among women worldwide, accounting for 23.8 and 15.8 %, respectively [1]. While Southeast Asia reports the lowest overall incidence of breast cancer globally, Thailand exhibits the highest rate within the region, followed by Singapore and Malaysia [2]. This contrast highlights the considerable burden of breast cancer in Thailand, suggesting the presence of localized factors that may influence disease prevalence[2]. Breast cancer treatment varies according to the tumour stage and may involve surgery, radiotherapy, endocrine therapy, targeted therapy, or adjuvant chemotherapy[3]. In stage IV breast cancer, treatment typically relies on systemic therapies such as chemotherapy, often in combination with targeted or immunotherapy, to manage metastases [4,5]. Surgery may be considered for palliative purposes in certain cases [3]. Access to standard breast cancer therapies, particularly chemotherapy for patients with advanced-stage disease, is frequently constrained by high costs [6]. This challenge is especially severe in rural areas of Thailand, where financial and healthcare resource limitations contribute to persistently low survival rates among breast cancer patients [7,8]. Therefore, there is an urgent need to explore and develop novel therapeutic agents derived from local natural resources, which may provide more accessible and cost-effective treatment alternatives for these patients.

Thailand is located in a tropical area with several critical natural resources worldwide, particularly *Mallotus* [9]. Several species within the *Mallotus* genus have long been utilized in traditional medicine across Southeast Asia to treat conditions such as liver disorders, skin ailments, fever, and malaria [10,11]. Compounds isolated from *Mallotus* extracts have demonstrated a range of bioactivities, such as antioxidant, antiviral, antimicrobial, anti-inflammatory, and cytotoxic effects [12,13]. Notably, a methanol extract derived from the leaves of *M. peltatus* has been evaluated for its activity against herpes viruses [14]. The ethanolic extract of red hairy substance from the fruits of *M. philippensis* showed strong antibacterial activity against selected gram-negative and gram-positive bacteria [15,16]. Finally, the leaf extract of *M. repandus* has been shown to possess analgesic and anti-inflammatory activities [17]. Moreover, *M. philippensis* and *M. repandus* have been demonstrated to exhibit anti-cancer properties against lung cancer cells, colorectal cancer cells, breast cancer cells, and cholangiocarcinoma cells [18,19]. However, although *M. glomerulatus* is a shrubby species found in northeastern and central Thailand [9], its phytochemical composition and biological activities remain largely unexplored, leaving its pharmacological potential poorly characterized. Therefore, local natural resources are the best option for urgent drug development to treat breast cancer patients in the country.

Based on the above information, it is hypothesized that *compounds isolated from M. glomerulatus* may be developed into effective treatment agents for breast cancer, reducing reliance on imported chemotherapy drugs, especially for those with limited financial resources in rural Thailand. This study aims to identify bioactive compounds that reduce breast cancer cell viability by screening natural products isolated from *M. glomerulatus*. The screening was conducted in both low-aggressiveness (MCF-7) and high-aggressiveness (MDA-MB-231) breast cancer cell lines [20-23] to evaluate differential efficacy across cancer subtypes. Given the critical role of vacuolar H^+^-ATPase (V-ATPase) in regulating lysosomal acidification, as well as its involvement in cancer progression [24], metastasis [25], and frequent overexpression in aggressive breast cancers [26,27], we hypothesized that active compounds from *M. glomerulatus* may exert their effects by targeting this proton pump. Therefore, V-ATPase was selected as the docking target to explore whether the isolated compounds could disrupt this cancer-associated mechanism and contribute to the observed cytotoxicity.

## Experimental

### General experimental procedures

Melting points were recorded (uncorrected) and determined on a digital Electrothermal Melting apparatus. Infrared spectra were recorded by using Perkin Elmer System 2000 FT-IR, whereas the major bands (*ν*_max_) were recorded in wave numbers, cm^-1^. Optical rotations were measured using a JASCO DIP-370 digital polarimeter with a 50 mm microcell (1 mL). Ultraviolet absorption spectra were measured in ethanol, chloroform, and methanol solutions on a JASCO 530 spectrophotometer. The principal bands (*λ*_max_) were reported as wavelengths and log *ε*. Low-resolution EI mass spectra were recorded at 70 eV (probe) on a Thermo Finnigan Polaris Q mass spectrometer. The high-resolution mass spectra (HRMS) were recorded on a Micromass model VQ-TOF2. The high-resolution nuclear magnetic resonance spectra were mainly recorded on Bruker AscendTM 400 and Bruker ADVANCE-500 spectrometers at the Department of Chemistry, Faculty of Science, Mahidol University. Solvents for extraction, chromatography, and recrystallization were distilled prior to use at their boiling point ranges. A pre-coated TLC aluminium sheet of silica gel 60 PF254 (20×20 cm, Merck) was used for analytical purposes, and the bands were visualized by ultraviolet light or anisaldehyde solution. Vacuum column chromatography was performed using silica gel 60H (230-400 mesh ASTM, Art. No. 7736, E. Merck), and column chromatography was performed using silica gel 60H (70-230 mesh ASTM, cat. No. 7734, E. Merck).

### Plant material

The aerial parts of *Mallotus glomerulatus* were collected in August 2011 from Tat Pho waterfall, Phu Langka National Park, Ban Phaeng District, Nakhon Phanom, Thailand. A voucher specimen (BKF 180558) has been deposited at the Forest Herbarium, Department of National Parks, Wildlife and Conservation, Bangkok, Thailand.

### Extraction and isolation of Mallotus glomerulatus

Dried and finely powdered aerial parts of *Mallotus glomerulatus* (4.4 kg) were percolated with MeOH (24 L×30 days×5 times) at room temperature to give a crude methanol (MeOH) extract (317.8 g). After dissolving in MeOH-EtOAc (1:1, 6 L) and solvent removal, the crude MeOH-EtOAc (1:1) soluble fraction (166.9 g) was separated by silica gel column (SiO_2_, 2 kg, CH_2_Cl_2_-hexane and MeOH-CH_2_Cl_2_ gradients) to give eight fractions (A1-A8). Fraction A3 (16.9 g) was fractionated using a silica gel column with an isocratic elution mixture of 10 % EtOAc in hexane as the eluent to obtain nine fractions (B1-B9). Fraction B1 (509.1 mg, eluted with 10 % EtOAc-hexanes) gave white colourless plates after recrystallization from EtOAc-hexanes, and was identified as friedelin (**3**). Fraction B4 (17.0 g, eluted with 20 % EtOAc-CH_2_Cl_2_) affords four fractions (C1-C4) after Si-gel CC. Fraction C3 (2.33 g) provided glomerulatusin (**2**) (863.0 mg) after recrystallization from EtOAc. Fraction C4 (3.32 g) was recrystallized from CH_2_Cl_2_ to afford anomaluone (**4**) (431.0 mg). Fraction B9 (2.13 g) yielded six fractions

(D1-D6) after Si-gel CC (20 % EtOAc-CH_2_Cl_2_). Fraction D1 (30.10 mg) provided pinostrobin (**7**) (21.1 mg) after recrystallization from MeOH. Fraction D3 (1.23 g) provided pinocembrin (**6**) (16.8 mg) after recrystallization from CH_2_Cl_2_. Fraction A5 (7.97 g, eluted with 70-90 % CH_2_Cl_2_-hexanes) was rechromatographed on a silica gel column (220 g, Merck Art. No. 7734, 5×20 cm), eluting with 20 % acetone-hexanes and finally with methanol. The solvents were evaporated to dryness, affording six fractions (E1-E6). Fraction E5 (3.40 g, eluted with 20 % acetone-hexanes) was rechromatographed on a Sephadex LH-20 column (113 g, 3×19 cm, 3 times), eluting with methanol to give three fractions (F1-F3). Fraction F2 (3.21 g, eluted with MeOH) was crystallized with MeOH-CH_2_Cl_2_ to give 5-hydroxy-6,7,3',4',5'-pentamethoxyflavone (**8**) (95.9 mg), as a yellow powder. The residue of fraction F2 (3.17 g) was rechromatographed on a silica gel column (113 g, Merck Art. No. 7734, 3×19 cm), eluting with 2 % MeOH-CH_2_Cl_2_ and finally with methanol. The solvents were evaporated to dryness, affording five subfractions (G1-G5). Fraction G3 (2.65 g) was crystallized with MeOH-CH_2_Cl_2_ to give 3,6-phenanthrenedione (**5**) (24.10 mg), as a colourless plate. Fraction G5 (382.0 mg) was obtained as green powder and identified as cleistanthin A (**10**) after recrystallization from EtOH. Fraction A7 (53.3 g, eluted with 10 to 40 % MeOH-CH_2_Cl_2_) was crystallized with methanol to give chrysin (**9**) (103.2 mg), as green powder. The residue of fraction A7 (51.92 g) was separated by column chromatography over silica gel, eluting with hexane and gradually enriching with CH_2_Cl_2_ in hexane until pure CH_2_Cl_2_ was obtained. This was followed by increasing amounts of methanol in CH_2_Cl_2_ and finally with pure methanol. The solvents were evaporated to dryness, affording four fractions (J1-J4). Recrystallization of Fraction J1 (2.11 g) from methanol afforded pinostrobin (**7**) (480.4 mg) and pinocembrin (**6**) (103.2 mg). Fraction J2 (3.17 g), eluted with 80 to 100 % CH_2_Cl_2_-hexanes, was subjected to column chromatography on silica gel using 20 % acetone-hexanes, followed by methanol, resulting in eight subfractions (K1-K8). Recrystallization of Fraction K8 (56.10 mg) from methanol afforded pure pinostrobin (**7**) (56.10 mg). Fraction J3 (23.75 g, eluted with 1to 3 % MeOH-CH_2_Cl_2_) was separated by column chromatography on silica gel, eluting with 50 % EtOAc-hexane and finally with methanol. The solvents were evaporated to dryness, affording four subfractions (L1-L4). Fraction L3 (9.34 g, eluted with 50 % EtOAc-hexanes) was crystallized with methanol to give a new compound, (3,4,5)-trihydroxy-6-methoxy-2,7-bis(3-methylbut-2-en-1-yl)-9*H*-xanthen-9-one (**1**) (186.2 mg).

### Cell culture

The human breast cancer cell lines (MCF-7 and MDA-MB-231) were purchased from ATCC (Manassas, VA, USA). MCF-7 cells were cultured in Dulbecco's Modified Eagle Medium/Nutrient Mixture F-12 (DMEM/F-12) (Gibco, New York, USA), which was supplemented with 10 % fetal bovine serum (FBS) (Gibco, New York, USA) and 1 % Penicillin-Streptomycin (P/S) (Gibco, New York, USA). MDA-MB-231 cells were cultured in DMEM high glucose (Gibco, New York, USA), supplemented with 10 % FBS and 1 % P/S.

### Primary and secondary screening of M. glomerulatus-isolated compounds against breast cancer cell lines

MCF-7 and MDA-MB-231 human breast cancer cell lines were seeded into 96-well plates (Corning, Acton, MA, USA) at a density of 10^4^ cells/well. MCF-7 cells were cultured in DMEM/F-12, while MDA-MB-231 cells were maintained in high-glucose DMEM. Both media were supplemented with 10 % foetal bovine serum (FBS) and 1 % penicillin/streptomycin (P/S). Cells were incubated for 24 h at 37 °C in a humidified atmosphere with 5 % CO_2_. For primary screening, test compounds isolated from *M. glomerulatus* were added to the culture medium at a final concentration of 50 μM. Doxorubicin was used as a positive control. Following compound treatment, cells were incubated for 72 h under standard culture conditions. After treatment, the medium was replaced with serum-free medium containing 0.5 mg mL^-1^ of 3-(4,5-dimethylthiazol-2-yl)-2,5-diphenyltetrazolium bromide (MTT), and cells were incubated for an additional 3 h. The MTT solution was then removed, and dimethyl sulfoxide (DMSO) was added to solubilize the formazan crystals. Absorbance was measured at 595 nm using a Microplate Spectrophotometer MR-850 BioPlate Reader (Biometrics Technologies, Wilmington, DE, USA). Compounds that reduced cell viability to ≤20 % compared to untreated controls were identified as hits for subsequent dose-response analysis. For secondary screening, hit compounds were tested at varying concentrations using two dilution schemes: a 10-fold series (100 to 0.00001 μM) and a 4-fold series (100 to 0.0061035 μM). Treated cells were incubated for 72 h, followed by an MTT assay as described above. The absorbance at 595 nm was recorded, and IC_50_ values were determined using GraphPad Prism 9 (GraphPad Software, San Diego, CA, USA).

### Cytotoxicity test in human liver cells

The human liver cells (HepG2 cells) were seeded into a 96-well plate at a cell density of 10^4^ cells/well and were incubated with DMEM/F-12 with 10 % FBS and 1 % P/S for 24 hours in an incubator (37 °C and 5 % CO_2_). After this incubation period, the effective compounds were added to the cell plate in a dose-dependent manner, mixing with culture media at concentrations of 100, 10, 1, 0.1, 0.01, 0.001, 0.0001, and 0.00001 μM. Then, they were additionally incubated for 72 hours. After 72 hours of incubation, the culture media containing the compounds were removed, and serum-free media containing MTT was added instead, with an additional 3 hours of incubation at 37 °C and 5 % CO_2_. After 3 hours of incubation, the MTT-containing media was removed and DMSO was added to the assay plates, resulting in different shades of purple colour measured by Microplate Spectrophotometer MR-850 BioPlate Reader. The calculation of CC_50_ was analysed by using GraphPad Prism 9.

### Molecular docking of (3,4,5)-trihydroxy-6-methoxy-2,7-bis(3-methylbut-2-en-1-yl)-9H-xanthen-9-one and cleistanthin A to Vacuolar H^+^-ATPase (V-ATPase)

The three-dimensional (3D) structure of yeast Vo V-ATPase (PDB ID: 7TAO [28]) was obtained from a previous study [29]. The chemical structure of cleistanthin A and novel xanthone ((3,4,5)-trihydroxy-6-methoxy-2,7-bis(3-methylbut-2-en-1-yl)-9H-xanthen-9-one) was drawn using PerkinElmer ChemDraw and subsequently converted into a 3D structure using Chem3D software. The resulting 3D structure was then energy-minimized using the Molecular Mechanics 2 (MM2) force field implemented in Chem3D. Following structure preparation, PDBQT files of both the protein and ligand were generated using the AutoDockFR 1.0 software suite [30]. Molecular docking was performed with AutoDock Vina version 1.2.7 [31]. The grid box was centred at coordinates *x* = 152.83, *y* = 171.45 and *z* = 133.06, with the exhaustiveness parameter set to 64; all other settings were maintained at default values. The best docking poses of cleistanthin A and xanthone bound to V-ATPase were visualized using UCSF ChimeraX [32], while 2D interaction diagrams of the protein–ligand complexes were generated using Discovery Studio Visualizer (BIOVIA, San Diego, CA, USA).

### Statistical analysis

Data analysis was performed using GraphPad Prism version 9 (GraphPad Software, San Diego, CA, USA). One-way analysis of variance (ANOVA) was used to compare values across multiple groups.

## Results

### Isolation and structural characterization of a new xanthone from M. glomerulatus

**Figure 1.**
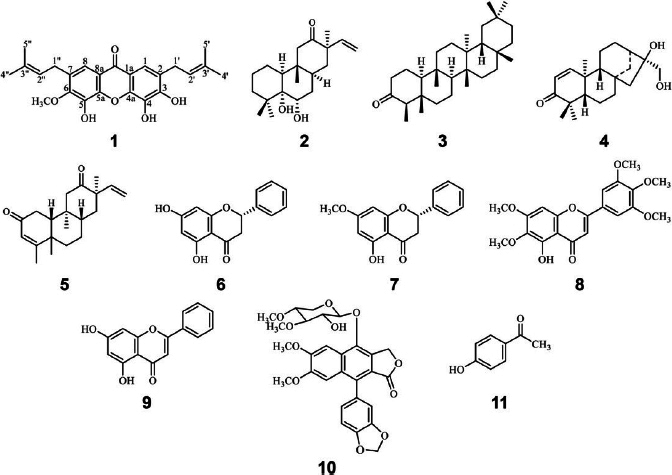
The structure of compounds **1** to **11** isolated from the *M glomerulatus*

*M. glomerulatus* is a shrubby species native to northeastern and central Thailand. Despite its regional presence, the phytochemical profile and biological properties of this species have remained largely unexplored. In the course of our phytochemical investigation, a new xanthone, (3,4,5)-trihydroxy-6-methoxy-2,7-bis(3-methylbut-2-en-1-yl)-9H-xanthen-9-one (**1**) (Figure 1), and a diterpenoid, glomerulatusin (**2**) [33,34], were isolated from the aerial parts of the plant.

Alongside these novel compounds, nine previously reported constituents were also identified: friedelin (**3**) [35,6], anomaluone (**4**) [33,37], 3,6-phenanthrenedione (**5**) [38], pinocembrin (**6**) [39,40], pinostrobin (**7**) [39,40], 5-hydroxy-6,7,3',4',5′-pentametho-xyflavone (**8**) [41], chrysin (**9**) [42], cleistanthin A (**10**) [43,44], and 4-hydroxyacetophenone (**11**) [45,46].

Compound **1** was obtained as a yellow powder by crystallization from MeOH, m.p. 178-180 °C. The molecular formula was determined as C_24_H_26_O_6_ by the high-resolution mass spectrometry (HR-ESI-MS) at *m*/*z* 433.1625, ([M+Na]^+^) (calcd. 433.1627 for C_24_H_26_O_6_Na). The EI mass spectrum showed the ions at *m*/*z* 411, [M+H]^+^, and 410, [M]^+^, which confirmed the molecular formula of **1**. The IR (KBr) spectrum showed the absorption bands at .max 3420 (O-H stretching of phenol), 2925 (aliphatic C-H stretching), 1644 (C=C stretching of alkene), 1609 (C=O stretching of ketone), 1583, 1455 (aromatic C=C stretching) and 1096, 1077, 1051 (C(O stretching of phenol) cm^-1^. The UV spectrum of 1 displayed three absorption bands at 243, 258, 316, and 354 nm. The 500 MHz ^1^H-NMR spectrum of this compound in MeOD (Table 1) exhibited a sharp singlet at *δ* 3.62 (3H, s, 6-OCH_3_), which was assigned to the methoxyl protons at position 6. The aromatic signals at *δ* 6.52 (1H, s, H-1) and *δ* 6.07 (1H, s, H-8) ppm were suggested to be the protons of a monosubstituted benzene ring in the molecule. The lack of other aromatic protons suggested that another aromatic ring was fully substituted. Two pairs of doublets at *δ* 3.91 (2H, d, 6.5 Hz, H-1′)/ 3.14 (2H, d, 7.2 Hz, H-1′′) and *δ* 1.55 (3H, d, 0.9 Hz, H-4′) / 1.54 (3H, br d, H-4′′), together with the broad doublets at *δ* 1.70 (3H, d, 0.7 Hz, H-5′) and 1.66 (3H, br d, H-5′′). The 125 MHz ^13^C-NMR spectrum of compound 1 (Table 1) showed twenty-four signals corresponding to twenty-four carbons. In combination with DEPT-135 spectrum, it was suggested to possess four methine carbons (*δ* 125.2, 123.9, 102.8 and 93.2 ppm), two methylene carbons (*δ* 27.1 and 22.2 ppm), two methyl signals for C-4′, 4′′ (*δ* 26.0 (CH_3_), 25.9 (CH_3_) ppm), two methyl signals for C-5′, 5′′ (*δ* 18.3 (CH_3_), 18.0 (CH_3_) ppm) and twelve quaternary carbons (*δ* 163.5, 161.5, 157.7, 156.6, 156.1, 144.7, 138.4, 131.7, 131.6, 112.3, 111.4 and 103.8 ppm)

**Table 1.** NMR data of compound 1 in MeOD-d4

Position	*δ*_H_, / ppm	*δ*_C_ / ppm	COSY correlated H	HMBC correlated C	NOESY Correlated H
1	6.52 (1H, s)	102.8, CH			8
1a		144.7, C		1, 1′	
2		112.3, C		1, 1′	
3		157.7, C		1	
4		138.4, C		1′	
4a		156.6, C		1	
5		161.5, C		1′′	
5a		156.1, C		8	
6		103.8, C		8	
7		163.5, C		8, 1′′	
8	6.07 (1H, s)	93.2, CH			
8a		111.4, C		8, 1′′	
9		183.1, C=O			
1′	3.91 (1H, d, *J* = 6.5 Hz)	27.1, CH2	2′, 4′, 5′		2′, 5′
2′	5.12-5.09 (1H, m)	125.2, CH	1′, 4′, 5′	1′, 4′, 5′	4′, 5′
3′		131.7, C		1′, 4′, 5′	
4′	1.55 (3H, d, *J* = 0.9 Hz)	26.0, CH3	1′, 2′, 5′	2′, 5′	
5′	1.70 (3H, d, *J* = 0.7 Hz)	18.3, CH3	1′, 2′, 4′	2′, 4′	
1′′	3.14 (1H, d, *J* = 7.2 Hz)	22.2, CH2	2′′, 4′′, 5′′		2′′, 5′′
2′′	5.12-5.09 (1H, m)	123.9, CH	1′′, 4′′, 5′′	1′′, 4′′, 5′′	6, 4′′, 5′′
3′′		131.6, C		1′′, 4′′, 5′′	
4′′	1.54 (3H, br d)	25.9, CH3	1′′, 2′′, 5′′	2′′, 5′′	5′′
5′′	1.66 (3H, br d)	18.0, CH3	1′′, 4′′, 4′′	2′′, 4′′	
6-OCH3	3.62 (3H, s)	61.3, OCH3			

**Figure 2.**
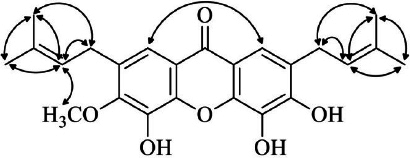
NOESY correlations observed of **1**

The skeleton of side chain was confirmed by the observed COSY correlations of H-1′/H-2′, H-4′, H-5′; H-2′/ H-1′, H-4′, H-5′; H-4′/H-1′, H-2′, H-5′; H-5′/H-1′, H-2′, H-4′; H-1′′/ H-2′′, H-4′′, H-5′′; H-2′′/ H-1′′, H-4′′, H-5′′; H-4′′/ H-1′′, H-2′′, H-5′′; H-5′′/ H-1′′, H-2′′, H-4′′; as well as the NOESY correlations of H-1/ H-8; H-2′′/ H-6, H-4′′, H-5′′ (Figure 2). Full assignments of ^1^H and ^13^C NMR signals were carried out based on 2D NMR analyses (Table 1).

The 500 MHz ^1^H-NMR spectrum of glomerulatusin (2) in CDCl3 exhibited four tertiary methyl groups at *δ* 1.32 (s, CH3-17), 1.26 (s, CH3-18), 1.02 (s, CH3-19), and 0.70 (s, CH3-20). The signals at *δ* 6.16 (dd, 17.7, 10.9 Hz, H-15), 5.04 (d, 17.7 Hz, H-16trans), and *δ* 5.13 (d, 10.9 Hz, H-16cis) were assigned to the olefinic protons at C-15 and C-16, respectively. A pair of rather deshielded methylene protons *δ* 2.39 (d, 13.9 Hz, H-11e) and 2.18 (d, 13.9 Hz, H-11a), indicated that these two hydrogen protons were in the para-position of a carbonyl group. The signal at *δ* 3.80 (dd, 11.8, 4.6 Hz, H-6) was assigned to the proton at carbon (C-6) carrying a hydroxyl group, the assignments of protons in structure 2, except the chemical shifts for H-17, H-18 and H-19, were found closely similar to those of anomallotusinin previously reported by Yang Yiping *et al.* [34]. The isolated compounds, along with the known constituents, were further evaluated for their activity against human breast cancer cell lines to explore their potential as anticancer agents.

### Two M. glomerulatus-derived compounds are effective against breast cancer cells MCF-7 and MDA-MB-231.

**Figure 3.**
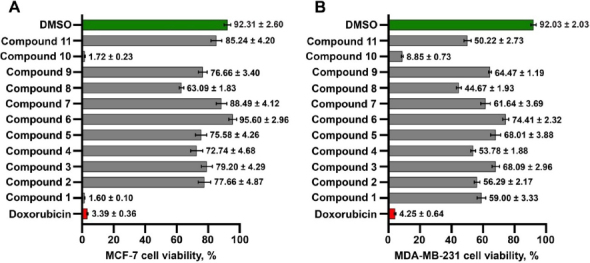
The primary screening, containing 11 compounds, was conducted in both MCF-7 (**A**) and MDA-MB-231 (**B**) breast cancer cell lines. The red color represents doxorubicin, a positive control, while the green color represents DMSO, a negative control. The grey colors are considered as the compounds extracted from *M. glomerulatus* including (3,4,5)-trihydroxy-6-methoxy-2,7-bis(3-methylbut-2-en-1-yl)-9*H*-xanthen-9-one (Compound **1**), glomerulatusin (Compound **2**), friedelin (Compound **3**), anomaluone (Compound **4**), 3,6-phenanthrenedione (Compound **5**), pinocembrin (Compound **6**), pinostrobin (Compound **7**), 5-hydroxy-6,7,3',4',5′-pentametho-xyflavone (Compound **8**), chrysin (Compound **9**), cleistanthin A (Compound **10**), 4-hydroxyacetophenone (Compound **11**). Cancer cells were treated with 11 compounds for 72 hours. Then, they measured the cell viability with the MTT assay. The percent cell viability was then analyzed and measured as a percentage. Data are shown as mean ± SEM from at least three bioreplicate independent experiments

The phytochemical properties and biological effects of *M. glomerulatus*, a natural resource primarily found in tropical regions, including Thailand, have not yet been thoroughly investigated. Therefore, exploring local natural resources presents a promising and urgent opportunity for developing new therapeutic agents for breast cancer patients within the country. In this study, 11 compounds isolated from *M. glomerulatus* were screened for their effects on breast cancer cell viability using the MTT assay. In the initial single-concentration screening (primary screening), two compounds, Compound 1 and 10, significantly inhibited the viability of MCF-7 (low-aggressiveness) cells by more than 80 %, while only Compound 10 achieved a similar inhibition in MDA-MB-231 (high-aggressiveness) cells (Figures 3A and 3B). Compound 1, which is a new xanthone and has been identified as (3,4,5)-trihydroxy-6-methoxy-2,7-bis(3-methylbut-2-en-1-yl)-9H-xanthen-9-one, showed strong selectivity toward MCF-7 cells, reducing viability to 1.60 ± 0.10 %. In contrast, Compound 1-treated MDA-MB-231 cells exhibited a viability of 59.00 ± 3.33 %. Notably, Compound 10, which has been identified as cleistanthin A, suppressed the viability of both MCF-7 and MDA-MB-231 cells by over 80 %. The efficacy of both hit compounds was comparable to that of the chemotherapeutic drug doxorubicin, 4.02 ± 0.24 % (MCF-7) and 4.72 ± 1.07 % (MDA-MB-231). These findings suggest that the two *M. glomerulatus*-derived compounds, 3,4,5-trihydroxy-6-methoxy-2,7-bis(3-methylbut-2-en-1-yl) and cleistanthin A, hold promise as potential anti-breast cancer agents. Therefore, both candidates will be further evaluated in secondary screening using dose-dependent assays to determine their IC_50_ values.

**Figure 4.**
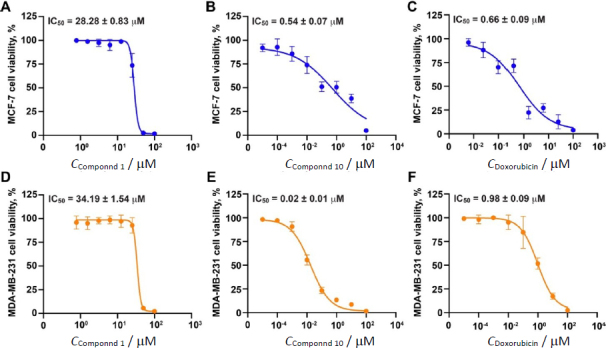
Dose-response effects (IC_50_ values) of hit compounds on breast cancer cells. (**A-C**) Dose-response effects on MCF-7 cell viability for compound **1** (**A**), compound **10** (**B**), and doxorubicin as a control (**C**). (**D-F**) Dose-response effects on MDA-MB-231 cell viability for compound **1** (**D**), compound **10** (**E**), and doxorubicin (**F**). Data are shown as mean ± SD. Notice that all of these statistical analyses were from at least three bioreplicate independent experiments

From the primary screening, two compounds isolated from *M. glomerulatus* were evaluated for their potential as anti-breast cancer agents in a dose-dependent manner, and IC_50_ values were calculated to determine their potency. Compound 1 exhibited moderate activity, with IC_50_ values of 28.28 ± 0.83 μM for MCF-7 cells and 34.19 ± 1.54 μM for MDA-MB-231 cells (Figures 4A and 4D).

However, its effectiveness was lower than that of doxorubicin, which showed IC_50_ values of 0.66 ± 0.09 μM and 0.98 ± 0.09 μM for MCF-7 and MDA-MB-231 cells, respectively (Figures 4C and 4F). Interestingly, Compound 10 demonstrated greater efficacy than doxorubicin, with significantly lower IC_50_ values of 0.54 ± 0.07 μM and 0.02 ± 0.01 μM for MCF-7 and MDA-MB-231 cells, respectively (Figures 4B and 4E). These findings suggest that cleistanthin A holds strong potential as an anti-breast cancer agent.

### Cleistanthin A acts as an anti-breast cancer agent without inducing cytotoxicity in liver cells

**Figure 5.**
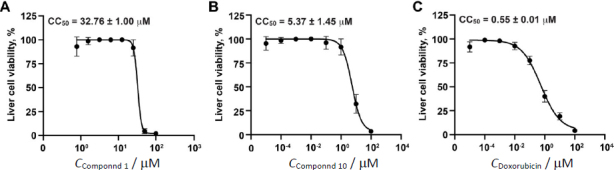
Cytotoxicity testing (CC_50_) of the hit compounds against the human liver cells. Dose-dependent cytotoxicity effects of compound **1** (A), compound **10** (B), and doxorubicin (C) on the viability of human liver cells (HepG2 cells)

The IC_50_ value of cleistanthin A plays a crucial role in evaluating its effectiveness as an anti-cancer agent, specifically in the treatment of breast cancer. Equally important, however, is the assessment of its cytotoxic concentration (CC_50_) in liver cells, which are the first cells to encounter and metabolize the compound. This evaluation helps define the therapeutic window of hit compounds, commonly expressed as the selectivity index (SI), which is the ratio of CC_50_ to IC_50_. A higher SI, which shows as 10 or higher, is preferable and indicates a greater margin of safety between the effective and toxic doses [47]. In this study, Compound **1** was found to have a concentration that causes toxicity close to the concentration that is active against breast cancer cells, with a CC_50_ of 32.76 ± 1.00 μM (Figure 5A). Furthermore, this new xanthone compound exhibits a lower therapeutic window, with an SI value of less than 10 (Table 2). Thus, Compound **1** was not studied further; however, it could be modified in the future to increase its efficacy and reduce its cytotoxicity. Meanwhile, Compound **10** exhibited a CC_50_ of 5.37 ± 1.45 μM in liver cells (Figure 5B), which is over two orders of magnitude higher than its IC_50_ values against breast cancer cells. This suggests a favorable safety profile. In comparison, doxorubicin, a standard chemotherapeutic drug, showed a CC_50_ of 0.55 ± 0.01 μM in liver cells (Figure 5C), indicating significantly higher toxicity. These results suggest that cleistanthin A is relatively safe for liver cells, which are likely to process the compound before it reaches its target tissues.

**Table 2.** Selectivity index (SI) of hit compounds in human liver cells

Compounds	IC_5_ / μM		CC_50_ / μM	SI (CC_50_/IC_50_)	
	MCF-7	MDA-MB-231	HepG2	HepG2: MCF-7	HepG2: MDA-MB-231
**1**	28.28	34.19	32.76	1.16	0.96
**10**	0.54	0.02	5.37	9.94	268.50
DoxorubICin	0.66	0.98	0.55	0.83	0.56

The SI values of the tested compounds were summarized in Table 2. Compound **10** exhibited SI values of approximately 10 for MCF-7 cells and over 100 for MDA-MB-231 cells, indicating a wide therapeutic window and strong selectivity toward breast cancer cells with minimal toxicity to liver cells. In contrast, doxorubicin showed SI values below 1 for both cell types, reflecting a very narrow therapeutic window and high liver cell toxicity. These findings emphasize the potential of Cleistanthin A as a safer and more selective anti-breast cancer agent compared to conventional chemotherapy. Its ability to selectively target cancer cells while sparing normal liver cells makes it a promising candidate for further preclinical and clinical development, particularly for treating advanced stages of breast cancer.

### Molecular docking and structural analysis of cleistanthin A and a new xanthone as potential V-ATPase inhibitors

**Figure 6.**
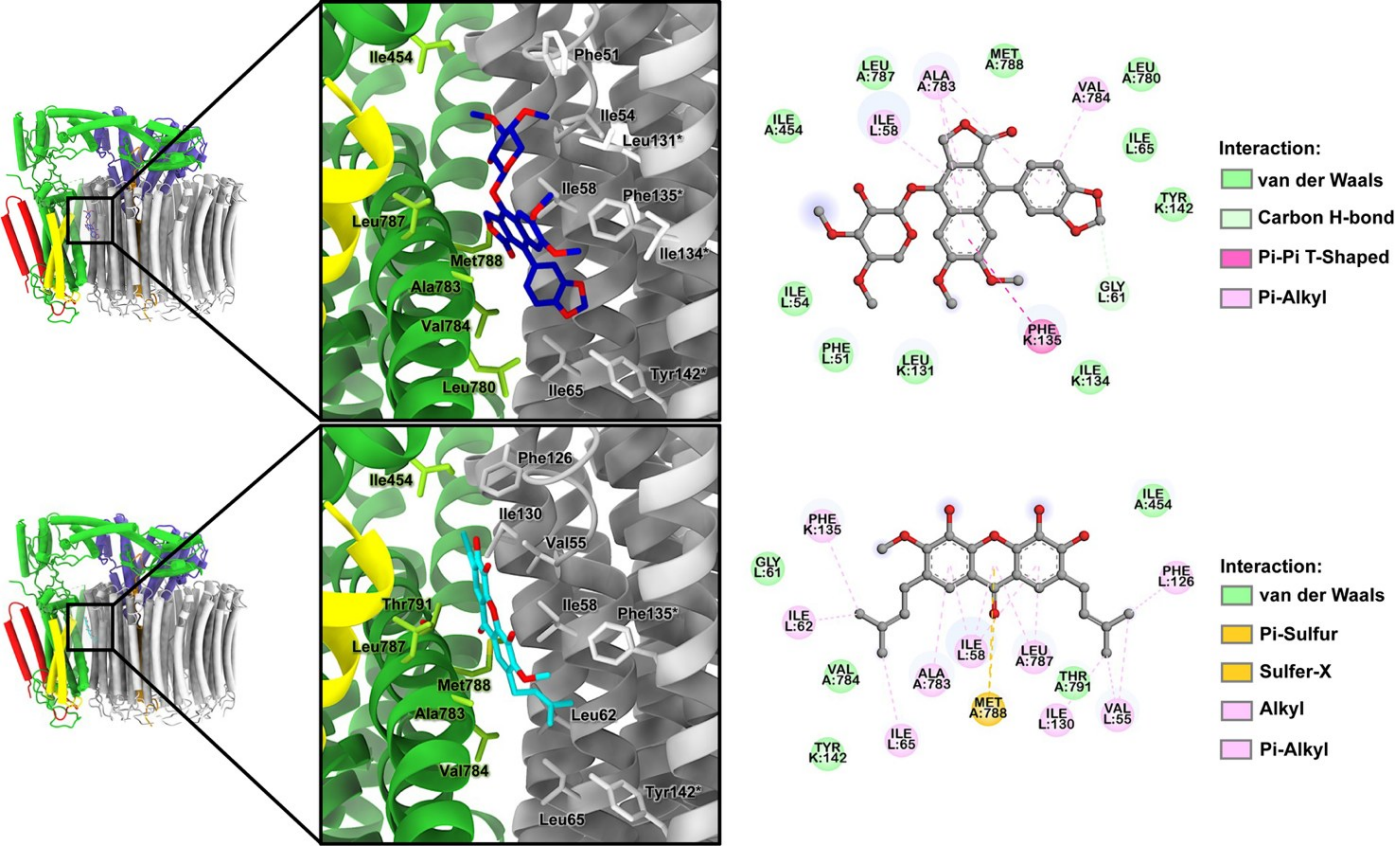
3D binding modes of cleistanthin A and xanthone at the interface between subunit a and two adjacent c subunits of VO V-ATPase, along with close-up views of the binding residues surrounding each compound, obtained from molecular docking (left panel). The binding interactions of cleistanthin A and xanthone with V-ATPase are further illustrated by 2D interaction diagrams (right panel). *Residues from the second (adjacent) c subunit

Cleistanthin A has been previously reported to exhibit anticancer activity through inhibition of V-ATPase, leading to lysosomal dysfunction and subsequent cancer cell death [29,48,49]. However, there have been no reports of such applications for breast cancer. In parallel, xanthones represent a diverse class of polyphenolic compounds commonly found in Garcinia species, many of which exhibit anticancer, antioxidant, and anti-inflammatory properties [50-53]. Moreover, xanthone derivatives, particularly garcinone E, have been previously shown to have promise as anticancer agents due to their ability to bind to and potentially inhibit V-ATPase[54]. Based on this evidence, it is hypothesized that *M. glomerulatus*-isolated xanthone (**1**) might also interact with and inhibit V-ATPase. This study aimed to investigate whether *M. glomerulatus*-isolated xanthone (**1**) could serve as a novel scaffold for V-ATPase inhibition, offering potential for future therapeutic development. To investigate the binding modes and affinities of cleistanthin A (**10**) and *M. glomerulatus*-isolated xanthone (**1**) toward vacuolar V-ATPase, a molecular docking study was conducted. Both compounds were docked into the interface between subunit a (shown in green in Figure 6) and two adjacent c subunits (shown in grey in Figure 6), targeting the same binding site previously used for docking the cleistanthin A derivative, ECDD-S18[29].

The docking results revealed that cleistanthin A (**10**) bound to V-ATPase with a binding energy of -29.3 kJ·mol^-1^ (-70 kcal·mol^-1^), while *M. glomerulatus*-isolated xanthone (**1**) exhibited a slightly stronger binding energy of -31.4 kJ·mol^-1^ (-75 kcal·mol^−1^). These findings suggest that both compounds have a comparable ability to bind V-ATPase, potentially disrupting the rotation of the c ring at the a–c interface and thereby inhibiting V-ATPase activity. Further analysis of the 2D interaction diagrams revealed that cleistanthin A (**10**) primarily engaged in π-alkyl interactions with Val783 and Val784 (subunit a) and Ile58 (subunit c), a π–π T-shaped interaction with Phe135 (adjacent c subunit), and non-classical carbon–hydrogen bonding with Gly61 (subunit c). Similarly, *M. glomerulatus*-isolated xanthone (**1**) formed multiple interactions with residues from subunits a and c. The *M. glomerulatus*-isolated xanthone (**1**) moiety established π-alkyl interactions with Ile58 (subunit c), Ala783, and Leu787 (subunit a), as well as π-sulphur and sulphur-X interactions with Met788 (subunit a). Additionally, the methylbutene groups located on both sides of the *M. glomerulatus*-isolated xanthone (**1**) structure engaged in π-alkyl interactions with Phe126 and Phe135, and alkyl interactions with Val55, Ile62, Ile65, and Ile130.

## Discussion

The phytochemical analysis of *Mallotus glomerulatus*, an underexplored member of the Euphorbiaceae family, resulted in the successful isolation and structural elucidation of a novel xanthone, (3,4,5)-trihydroxy-6-methoxy-2,7-bis(3-methylbut-2-en-1-yl)-9*H*-xanthen-9-one. This compound represents the first xanthone reported from *M. glomerulatus*, thereby contributing new structural data to the limited phytochemical record of this endemic Thai species [9,12,33]. The presence of a xanthone framework within this plant genus is of particular interest, as such structures have been predominantly reported in unrelated genera such as *Garcinia*, *Swertia*, and *Calophyllum*, where they exhibit significant biological activities [50,52]. The structural features of the new compound were thoroughly confirmed using modern spectroscopic techniques, including UV, IR, 1D and 2D NMR, and high-resolution mass spectrometry. The IR spectrum showed characteristic signals for phenolic hydroxyl (3420 cm^-1^), aromatic and aliphatic C–H stretching, and conjugated C=O (1609 cm^-1^), consistent with xanthonoid structures previously isolated from medicinal plants[53]. UV absorption maxima at 243, 258, 316 and 354 nm further supported the existence of a highly conjugated aromatic system typical of polyhydroxylated xanthones[52]. The ^1^H and ^13^C NMR spectra revealed a symmetric substitution pattern: hydroxyl groups at positions C-3, C-4, and C-5, and a methoxy group at C-6 on ring A, with two prenyl (3-methylbut-2-en-1-yl) side chains symmetrically located at C-2 and C-7. This arrangement closely resembles the substitution patterns found in cytotoxic xanthones, such as α-mangostin and garcinone E, both of which demonstrate high selectivity toward tumour cells[51,54].

The novel prenylated xanthone (Compound **1**) and cleistanthin A (Compound **10**) exhibited significant cytotoxicity against breast cancer cell lines. Compound 1 showed pronounced activity against MCF-7 cells, consistent with the literature, which demonstrates that prenylated xanthones, especially those with C-2 prenylation, possess enhanced efficacy against ER-positive tumors by increasing lipophilicity, membrane permeability, and possibly modulating the estrogen receptor. For instance, prenylated xanthones such as those from *Garcinia mangostana* and *Garcinia mckeaniana* have demonstrated nM-range IC_50_ values in both ER-positive (MCF−7) and triple-negative (MDA-MB-231) breast cancer lines [55-59]. Cleistanthin A exhibited broad-spectrum cytotoxicity against both MCF−7 and MDA−MB−231, in line with previous observations from colorectal, cervical, melanoma, and head-and-neck cancer cell lines, where apoptosis, mitochondrial dysfunction, and V−ATPase inhibition were key mechanisms [48,49,60]. The dose-response experiments demonstrated that cleistanthin A has IC_50_ values in the low-nanomolar range, surpassing the efficacy of the xanthone and doxorubicin. This finding is consistent with Wongpan *et al.* [60], who reported a similar nanomolar potency for cleistanthin derivatives in head and neck cancer cells via V−ATPase targeting. By contrast, the novel xanthone had mid-micromolar IC_50_ values; this aligns with established trends in xanthone research, where non-optimized derivatives typically display moderate cytotoxicity unless modified for improved pharmacological performance [57,59,61-64].

The biological evaluation of compound **1** and compound **10** against two phenotypically distinct breast cancer cell lines, MCF-7 and MDA-MB-231, demonstrates their differential but promising anticancer potential. MCF-7 is a luminal A subtype, estrogen receptor-positive (ER^+^), and considered less aggressive, while MDA-MB-231 represents the triple-negative breast cancer (TNBC) subtype, characterized by the absence of ER, PR, and HER2 expression, and is associated with higher aggressiveness, invasiveness, and poor prognosis [65,66]. Compound **1**, a newly identified prenylated xanthone, exhibited selective cytotoxicity against MCF-7 cells. This result is consistent with previous studies, which have shown that xanthones, particularly those with prenyl or hydroxyl substitutions, demonstrate enhanced activity in ER^+^ breast cancer cells by modulating oestrogen signalling, mitochondrial function, or inducing oxidative stress [50,54]. The selective activity of compound 1 suggests its potential as a therapeutic candidate for hormone-responsive, low-aggressiveness breast cancers, where treatment options are often successful but long-term resistance remains a challenge. Its distinct structural features, especially the bis-prenylated aromatic core, may allow further chemical optimization to broaden its activity spectrum or increase potency. In contrast, compound **10** (cleistanthin A) demonstrated broad-spectrum cytotoxicity, with strong efficacy in both MCF-7 and MDA-MB-231 cells. This finding is significant, as TNBC remains one of the most difficult subtypes to treat due to its heterogeneity, lack of hormonal targets, and resistance to conventional chemotherapy[66,67]. The ability of cleistanthin A to inhibit proliferation in both cell types implies a mechanism that bypasses receptor dependency. Indeed, prior studies have confirmed that cleistanthin A and related diphyllin-type lignans inhibit the V-ATPase, disrupt lysosomal acidification, and impair autophagy, a pathway commonly upregulated in TNBC to sustain survival under metabolic stress [60,68]. Thus, cleistanthin A holds therapeutic potential as a non-receptor-targeted agent capable of suppressing both indolent and aggressive breast cancer phenotypes.

Although the exact mechanism of action in breast cancer cells remains unclear, emerging evidence suggests that the compound may exert its anticancer effects by targeting vacuolar H^+^-ATPase (V-ATPase). V-ATPase is a proton pump involved in maintaining intracellular pH and lysosomal acidification, processes that are critical for cancer cell survival and progression [24,69]. In cancer, V-ATPase activity is frequently upregulated, contributing to tumour invasiveness, metastasis, and chemoresistance by acidifying the tumour microenvironment and facilitating the sequestration of lysosomal drugs [25]. Consequently, V-ATPase has emerged as a promising therapeutic target in oncology. V-ATPase plays a central role in the autophagic pathway, particularly in the fusion of autophagosomes with lysosomes and the subsequent degradation of cellular components [70,71]. Inhibition of V-ATPase can block autophagic flux, leading to the accumulation of autophagosomes and impaired cellular clearance. This autophagy flux inhibition can promote stress-induced cell death, especially in cancer cells that rely on autophagy for survival under harsh microenvironments [49]. Therefore, the *M. glomerulotus*-isolated compounds may exert their cytotoxic effects in breast cancer cells by inhibiting both V-ATPase activity and autophagic flux, ultimately leading to reduced cell viability and cancer cell death.

The molecular docking analysis revealed that both cleistanthin A and the novel xanthone exhibited favourable binding affinities toward the V-ATPase enzyme, particularly at the interface between subunit a and the adjacent c subunits. Cleistanthin A showed a docking score of -29.3 kJ·mol^-1^ (-70 kcal·mol^-1^), which is consistent with previous studies demonstrating that diphyllin-type lignans exert anticancer effects through inhibition of V-ATPase, a proton pump critical for maintaining the acidic environment of intracellular organelles such as lysosomes and endosomes [48,60] The ability of cleistanthin A to interact with key residues such as Val783, Val784, and Ile58 suggests that the compound may disrupt proton translocation and interfere with organelle acidification, mechanisms that have been shown to lead to cancer cell apoptosis and autophagy inhibition [49,60]. The xanthone compound also demonstrated a strong binding affinity, with a docking score of -31.4 kJ·mol^-1^ (-75 kcal·mol^-1^), indicating a comparable potential to inhibit V-ATPase activity. The structural features of the xanthone, namely the hydroxylated aromatic rings and bis-prenylated side chains, contributed to multiple interactions with hydrophobic residues such as Ile62, Ile130, and Leu787, as well as π-alkyl and π-sulphur interactions with Met788. These interactions are indicative of stable ligand–protein complexes, which are essential for effective enzyme inhibition. Previous reports have shown that polyphenolic scaffolds, particularly xanthones, can interfere with V-ATPase function and induce lysosomal dysfunction in cancer cells [50,54]. The docking results support this mechanism, suggesting that the novel xanthone may act through a similar inhibitory pathway. The presence of π–π T-shaped interactions between both compounds and residues such as Phe126 and Phe135 further supports their potential to anchor at the a–c subunit interface of the enzyme. This region has been identified as a critical target site for V-ATPase modulation, as previously described in structural studies using cryo-EM and small-molecule inhibitors[28]. The ability of both cleistanthin A and the xanthone to occupy this region reinforces their candidacy as V-ATPase-targeting agents. While cleistanthin A has been experimentally validated in multiple cancer models for its V-ATPase-dependent cytotoxicity, the xanthone’s predicted binding profile provides a strong rationale for further in vitro and in vivo evaluation.

The presence of bis-prenylated moieties in xanthones has been associated with enhanced anticancer potency, attributed to increased lipophilicity, cell permeability, and binding to the hydrophobic regions of protein targets [50]. For instance, the prenylation on the xanthone skeleton significantly improves anticancer efficacy by facilitating membrane interaction and target specificity[50]. Furthermore, the garcinone E, which features a similar substitution pattern, could inhibit autophagy in ovarian cancer cells by disrupting lysosomal function through V-ATPase inhibition [54]. These parallels support the hypothesis that the novel xanthone from *M. glomerulatus* may share a similar mechanism of action. Cleistanthin A, a diphyllin-type arylnaphthalene lignan, possesses a structural architecture that supports effective intracellular delivery and target engagement, particularly relevant to its proposed mechanism of V-ATPase inhibition. Its molecular features include multiple methoxy groups, a methylenedioxy bridge, and a lactone ring, which collectively contribute to moderate lipophilicity and favourable passive permeability [72-75]. Additionally, its molecular weight, approximately 540.5 g mol^-1^, slightly exceeds the Lipinski rule threshold (500 Da) [76,77], but remains within the acceptable range observed for many bioactive natural products [77,78]. Several clinically approved anticancer drugs, including taxanes and epipodophyllotoxins, exceed 500 Da but retain oral bioavailability due to structural rigidity and efficient transporter engagement[79]. Molecular docking results from this study revealed that cleistanthin A binds favourably to the a–c subunit interface of vacuolar H^+^-ATPase, with a docking score of -29.3 kJ·mol^-1^ (-70 kcal·mol^-1^). The compound forms multiple π-alkyl and hydrophobic interactions with key transmembrane residues, including Val783, Val784, Ile58, and Phe135, regions associated with proton transport and enzyme function [49]. These findings are consistent with previous studies reporting that lipophilic natural products often anchor within the hydrophobic transmembrane domains of V-ATPase and similar proton pumps [28,80,81]. Structurally, cleistanthin A's planar aromatic naphthalene core facilitates π–π stacking, while the methylenedioxy and methoxy substituents enhance its compatibility with lipophilic protein cavities [52]. The physicochemical profile of cleistanthin A supports its candidacy as a cell-permeable anticancer agent that acts by V-ATPase inhibition. Previous work has demonstrated that diphyllin-based lignans, such as cleistanthin A, preferentially accumulate in acidic intracellular compartments, such as lysosomes, due to passive pH-dependent diffusion and V-ATPase binding, leading to autophagy blockade and cancer cell apoptosis [49,82]. Importantly, these properties contribute not only to its cytotoxic selectivity against cancer cells but also to its low toxicity in non-malignant cells, including hepatocytes, which typically maintain a neutral intracellular pH [47].

Although the HepG2 cells are a hepatoma cell line, they are commonly used for pharmacotoxicological research. It is not only a nontumorigenic and highly proliferative cell, but it also offers several benefits such as high availability, easy to handle, almost unlimited life-span, and stability [83] In HepG2 liver models, cleistanthin A showed negligible cytotoxicity, yielding a selectivity index >100 in MDA−MB−231 cells and 210 in MCF−7, mirroring reports that diphyllin-type lignans preferentially accumulate in acidic tumours and spare non-malignant tissues likewise reported low hepatocyte toxicity at similar concentrations [68,84,85]. By contrast, doxorubicin induced notable liver cell toxicity, consistent with well-established dose-limiting hepatotoxicity in clinical use [47]. The novel xanthone displayed limited selectivity (SI <10), suggesting the need for structural modifications such as glycosylation to enhance safety and pharmacokinetic properties, strategies previously shown to improve xanthonoid profiles [52,86].

The dual effectiveness of these two structurally unrelated compounds reflects complementary benefits: compound **1** may serve as a targeted option for early-stage, hormone-sensitive tumours, while compound **10** offers broad applicability across subtypes, including aggressive and treatment-resistant TNBC. Moreover, the low hepatotoxicity of cleistanthin A observed in HepG2 cells further supports its candidacy as a lead compound with a favourable therapeutic index, which is especially important in advanced disease settings where long-term systemic toxicity is a concern [68,84]. Together, these findings underscore the therapeutic value of *Mallotus glomerulatus*-derived compounds and justify further mechanistic and *in vivo* studies to validate their efficacy and safety.

## Conclusions

This study highlights the therapeutic potential of two phytochemicals isolated from Mallotus glomerulatus, a novel xanthone (compound **1**) and cleistanthin A (compound **10**), against breast cancer. Compound **1** exhibited selective cytotoxicity toward the low-aggressive MCF-7 breast cancer cells, whereas cleistanthin A demonstrated potent, broad-spectrum cytotoxicity against both the low-aggressive MCF-7 and the highly aggressive MDA-MB-231 cells. Dose-response studies confirmed that cleistanthin A possesses nanomolar-range IC_50_ values and a high selectivity index, indicating strong efficacy and low toxicity toward normal liver cells. Molecular docking further revealed that both compounds interact with critical hydrophobic residues at the a–c subunit interface of V-ATPase, suggesting a shared mechanism of action through proton pump inhibition, which is known to suppress tumour survival pathways such as autophagy.

Despite these promising results, several limitations remain. Future research may focus on validating these hit compounds, performing mechanistic studies to confirm V-ATPase inhibition, and conducting medicinal chemistry efforts to enhance the bioactivity and safety profile of the novel xanthone scaffold. Overall, *M. glomerulatus*-derived compounds, especially cleistanthin A, represent promising leads for the development of affordable and effective anti-breast cancer therapies.
